# Characteristics of transgender health-related provider-to-provider consultations in Brazil’s public primary health care system: A cross-sectional study from a Southern Brazilian telehealth service

**DOI:** 10.20945/2359-4292-2026-0086

**Published:** 2026-08-05

**Authors:** Geferson Pelegrini, Fabiana Carvalho, Dimitris Rucks Varvaki Rados, Tayane Muniz Fighera, Natan Katz, Roberto Nunes Umpierre, Marcelo Rodrigues Gonçalves

**Affiliations:** 1 Programa de Pós-Graduação em Epidemiologia, Faculdade de Medicina, Universidade Federal do Rio Grande do Sul (UFRGS), Porto Alegre, RS, Brasil; 2 TelessaúdeRS, Porto Alegre, RS, Brasil; 3 Programa de Pós-Graduação em Endocrinologia, Faculdade de Medicina, Universidade Federal do Rio Grande do Sul (UFRGS), Porto Alegre, RS, Brasil; 4 Departamento de Medicina Interna, Faculdade de Medicina, Universidade Federal do Rio Grande do Sul (UFRGS), Porto Alegre, RS, Brasil; 5 Departamento de Medicina Social, Faculdade de Medicina, Universidade Federal do Rio Grande do Sul (UFRGS), Porto Alegre, RS, Brasil; 6 Hospital de Clínicas de Porto Alegre (HCPA), Porto Alegre, RS, Brasil

**Keywords:** Telemedicine, remote consultation, transgender persons, health services for transgender persons, primary health care

## Abstract

**Objective:**

Transgender individuals often face structural barriers and adverse health outcomes. Provider-to-provider (P2P) consultations may improve the quality of care for this population. This study describes the characteristics, clinical questions, and care provided to transgender population in the Brazilian public primary health care (PHC) system, using data from a P2P telephone consultation service based in Rio Grande do Sul State.

**Subjects and methods:**

This cross-sectional study included all consultations classified as related to transgender health care conducted between TelessaúdeRS consultants and PHC professionals from 2015 to 2023. We extracted patient characteristics and clinical questions from each consultation. Continuous data are reported as means ± standard deviations or medians (interquartile ranges), and categorical data as absolute and relative frequencies.

**Results:**

Between 2015 and 2023, 752 of the 422,972 total consultations were classified as related to transgender health care. Regarding sex assigned at birth, 47.3% of patients were male, 43.4% were female, and 9.3% were unreported. The mean patient age was 24.5 years (±9.1). The most frequent consultation topics were laboratory monitoring (64.9%), hormone therapy (49.7%), and mental health (28.9%). Nonprescription hormone use was reported by 41.0% of patients. Referrals to specialized care were recommended in 68.4% of cases, and clinical guidance was provided in 71.4%.

**Conclusion:**

The growing number of P2P consultations reflects an increasing demand for support regarding transgender care within PHC. Frequently asked questions reveal knowledge gaps among PHC providers, and the high rate of unsupervised hormone use suggests persistent barriers to appropriate care. P2P consultations can support PHC professionals in providing safer, more timely transgender health care.

## INTRODUCTION

The global prevalence of self-identified transgender individuals is estimated at approximately 0.6%, though estimates vary by region, age, and study method (^1^). In Brazil, recent estimates suggest a prevalence of 0.69% among adults, with relatively uniform distribution across regions (^2^). The transgender population faces significant barriers to accessing public health services, including stigma, discrimination, and geographic inequities (^3^). These barriers underscore the urgent need for inclusive and equitable public health policies.

The demand for comprehensive gender-affirming care for transgender individuals has increased significantly, while the average age at treatment initiation has decreased by approximately four years (^4^). Moreover, care-seeking among individuals assigned female at birth (AFAB) has increased substantially, now equaling or surpassing that of those assigned male at birth (AMAB), including transgender, non-binary, and other gender-diverse individuals (^4^-^6^). Although these trends are encouraging and may reflect greater societal acceptance (^4^), they also present new challenges for healthcare systems that are often unprepared to meet this rising demand.

Despite being limited, existing literature indicates that specialized healthcare services and hospital-based procedures are concentrated in large urban centers and more socioeconomically developed regions (^3^,^6^-^9^). Limited access to care is frequently associated with nonprescribed hormone use and high rates of suicidal ideation prior to gender transition (^6^,^10^,^11^). As primary health care (PHC) often serves as the main entry point into the health system, transgender individuals frequently rely on these services (^12^). However, insufficient training in gender-affirming care, uncomfortable patient-provider interactions, and systemic discrimination within healthcare settings limit the effectiveness of care (^7^,^10^,^13^).

There is growing evidence supporting the utility of remote consultations, offered in both patient-directed and provider-to-provider (P2P) modalities (^14^-^16^). Such consultations have shown benefits in the management of stigmatized conditions, including syphilis in pregnancy, tuberculosis, and psychiatric disorder (^17^-^20^). Additional advantages include reduced travel time and lower costs (^21^). Thus, the use of telehealth to support transgender care represents a promising opportunity, particularly given the challenges PHC professionals face in this domain. This study aimed to describe the characteristics, clinical questions, and care provided to transgender individuals within Brazil’s public PHC system, utilizing data from a large P2P telephone consultation service.

## SUBJECTS AND METHODS

### Study design and settings

**Figure 1:**
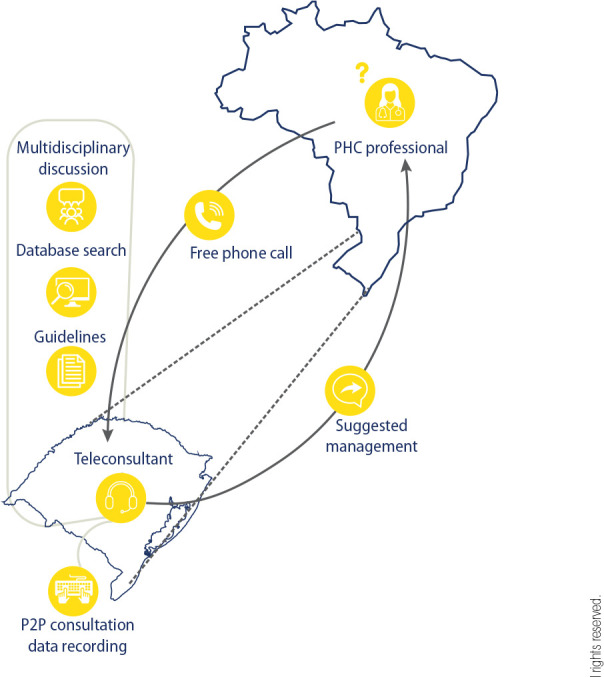
TelessaúdeRS synchronous provide-to-provider telephone consultation service.

PHC: primary health care; P2P: provider-to-provider.

This cross-sectional retrospective study analyzed records from a telehealth service and adhered to the STrengthening the Reporting of OBservational studies in Epidemiology (STROBE) guidelines (^22^). Data were obtained from TelessaúdeRS, a toll-free P2P telephone consultation service supporting public PHC providers in Brazil, based in Rio Grande do Sul State. The service offers telehealth support during or after patient encounters, including consultation, diagnosis, gatekeeping, and continuing education for physicians, nurses, and dentists working in PHC nationwide. It operates on business days from 8:00 am to 6:00 pm, enabling real-time consultations between PHC professionals and specialist consultants (**Figure 1**).

Data were collected from 2015 to 2023 and extracted from the TelessaúdeRS database (Research Organization Registry [ROR]: https://ror.org/00g229b55). Each consultation was initiated by a PHC professional and recorded in a standardized report stored on a secure university server. Consultants reviewed the clinical question and available resources and provided evidence-based recommendations based on TelessaúdeRS clinical guidelines and national protocols (^17^). Although they did not have specific training in transgender health, consultants engaged in interdisciplinary discussions and followed the same evidence-based processes. Each consultation concluded with a proposed care plan and guidance on the appropriate level of care (i.e., PHC, specialized service, or emergency referral).

### Participants

We selected consultations from January 2015 to December 2023 in which the primary topic was health care for the transgender population. Consultations focusing on clinical questions or guidance related to transgender health were included. Consultations that were misclassified (unrelated to transgender health), duplicated, or incomplete were excluded. Data were accessed for research purposes in August 2024 and analyzed between September and November 2024. For patients discussed in multiple consultations, the first call was defined as the index consultation, and subsequent calls were classified as follow-up discussions.

### Variables

We reviewed each consultation was reviewed to extract patient, clinical, PHC professional, and consultant data. For patients, variables included age (years), sex assigned at birth (male or female), reported comorbidities, age of self-identification with gender diversity, history of hormone therapy (categorized as none, nonprescribed, or prescribed), type of hormones used in nonprescribed (classified as nonor guideline-recommended formulations), receipt of mental health support, type of mental health service accessed (psychologist, psychiatrist, or public health system), and presence of a support network, defined by the presence of a responsible adult during the consultation for patients under 16 years of age.

For PHC professionals and consultants, gender (male or female), professional category, and age were recorded. Patients were stratified into two age groups: < 16 and ≥ 16 years, based on the age criterion for hormone therapy initiation in Brazil. From the consultation records, we also extracted information regarding the type of consultation (specialist referral, clinical discussion, or both), mean age at referral, main clinical discussion topics, adherence to recommendations provided (categorized as yes, no, or partial), presence of vulnerability conditions, cancer screening status, and geographic location (state and municipality) of the consultation.

Not all variables were documented in every consultation, as certain data were not always pertinent to the clinical question. When this occurred, variables were recorded as unreported. These missing data did not compromise the descriptive nature of the analysis. The primary objective was to describe the characteristics of patients and their consultations; no *a priori* outcomes were defined.

### Data analysis

Continuous variables were summarized as means with standard deviations (SD) (mean ± SD) or medians with interquartile ranges (median [Quartile_25-75_]), while categorical variables were reported as absolute and relative frequencies. No comparative or inferential statistical analyses were planned. Data analysis was performed using Microsoft Excel and R software (version 4.3.1). A convenience sample was used, comprising all eligible telephone consultations during the study period.

### Ethical approval

The study was approved by the Ethics Committees of *Hospital de Clínicas de Porto Alegre* (CAA no. 62602522.9.0000.5327) and AGHUse (protocol no. 2022-0371). As anonymized data from the TelessaúdeRS database were utilized, informed consent was waived.

## RESULTS

**Figure 2:**
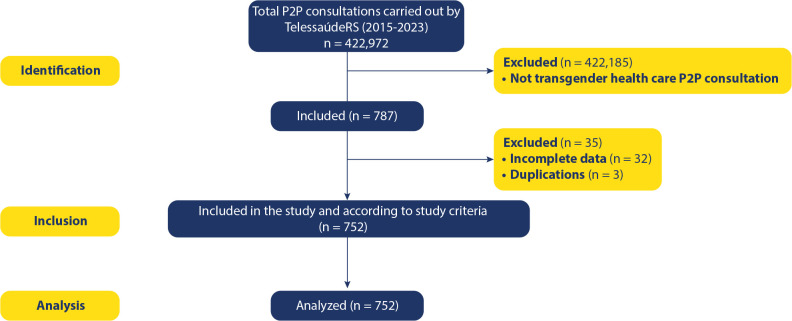
Flowchart of provider-to-provider telephone consultations about transgender health care carried out by TelessaúdeRS (2015-2023).

Of the 422,972 consultations conducted by TelessaúdeRS over the study period, 787 were classified as related to transgender health care. After excluding 35 consultations due to duplication or incomplete data, 752 were included in the analysis (**Figure 2**). Among these patients, 47.3% were AMAB and 43.4% AFAB, indicating a relatively balanced distribution. The mean age at consultation was 24.5 years (SD ± 9.1), with a median of 22 years (18; 28). **Table 1** summarizes the age distribution and additional patient characteristics.

**Table 1:** Characteristics of transgender patients in TelessaúdeRS provider-to-provider telephone consultations (2015-2023)

Clinical characteristic - Transgender patients		N (%) or mean ± standard deviation
Age at consultation (years) n = 702^a^	< 16 years ≥ 16 years	54 (7.7) 648 (92.3)
Sex assigned at birth n = 682^a^	Male Female	356 (52.2) 326 (47.8)
Reported comorbidities n = 179^a^	Depression Smoking General anxiety disorder HIV Hypertension	38 (21.2) 36 (20.1) 26 (14.5) 20 (11.2) 15 (8.4)
Age of self-identification as transgender n = 47^a^		12.6 ± 3.81
Hormone therapy history n = 471^a^	Without prior use Prescribed hormone use Non prescribed hormone use	157 (33.3) 118 (25.1) 196 (41.6)
Nonprescribed hormone use n = 196^a^	AMAB (n = 109)	
	Non-guideline recommended formulation Prioritized antiandrogenic formulations Prioritized estradiol formulations	90 (82.6) 36 (^33^) 25 (22.9)
	AFAB (n = 62)	
	Guideline recommended testosterone formulations Contraceptives/hormones to stop menstruation Non-guideline recommended testosterone formulations	53 (85.5) 10 (15.1) 1 (1.6)
Mental health support n = 166^a^	Psychologist follow-up Psychiatric follow-up Public health system mental health care	64 (38.5) 40 (24.1) 47 (28.3)

a Unreported data are not shown in the table. The sample size (n) for each variable is presented in the first column, where applicable. AMAB: assigned male at birth; AFAB: assigned female at birth; HIV: human immunodeficiency virus infection.

P2P: provider-to-provider.

Most individuals were receiving some form of hormone therapy, frequently through nonprescribed use. Among AMAB patients, non-guideline-recommended formulations were most commonly reported. In transgender men already undergoing appropriate hormonal monitoring, isolated progestins were used as a contraceptive method. For individuals who had not yet initiated testosterone therapy, combined oral contraceptives were frequently prescribed in PHC settings to suppress menstruation.

The mean age of PHC professionals was 37.6 years (SD ± 10.7; n = 295), whereas that of consultants was 34.7 years (SD ± 4.2; n = 394). Female professionals accounted for 67.1% of PHC providers (n = 695) and 71.4% of consultants (n = 707). Among PHC professionals (n = 690), 95.6% of consultations were conducted by physicians, of whom 83.0% were general practitioners without residency training, 9.6% were family physicians, and 3.0% represented other specialties. Nurses and psychologists participated in a smaller proportion of consultations.

Most consultations (61.8%; n = 436) were answered by generalist medical professionals (e.g., family medicine or internal medicine), while 23.9% (n = 169) were handled by endocrinologists, and 10.8% (n = 76) by other specialists. In addition, 3.5% (n = 25) of consultations were conducted by nurses. Consultations were conducted across Brazil, with participation from 12 of the 27 states. Although the service is available nationwide, the vast majority (88%, n = 662) originated in Rio Grande do Sul, where the service is based.

**Figure 3:**
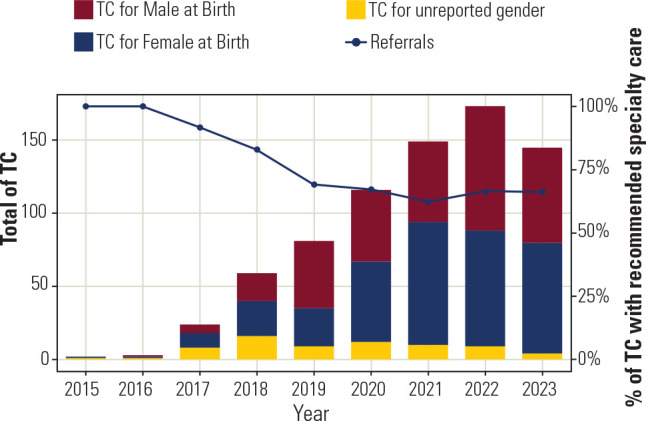
Annual number of provider-to-provider telephone consultations related to transgender care and proportion of recommended referrals, stratified by sex assigned at birth, in Brazil’s primary health care system.

The number of transgender-related case discussions increased steadily over the years. The first consultation occurred in 2015, two years after service initiation. The peak was in 2022, with 173 consultations, representing 23% of the study sample. **Figure 3** presents the annual distribution of consultations and referral rates. Overall, 68.4% of consultations resulted in referrals, and clinical guidance was provided in 71.4%. **Table 2** outlines the consultation topics and corresponding recommendations. Most consultations involved both clinical and referral-related questions. The most frequently addressed clinical issues were monitoring exams and hormone therapy. A substantial proportion also focused on broader aspects of patient evaluation, including clinical history and mental health concerns. In consultations with follow-up data, recommendations were generally followed. PHC professionals or consultants rarely assessed social vulnerability conditions.

**Table 2:** Management characteristics of provider-to-provider consultations concerning transgender health provided by TelessaúdeRS

Clinical inquiries from PHC professionals		N (%)
P2P consulting recommendation	Clinical discussion and case referral Case referral Clinical discussion	263 (35.0) 251 (33.4) 238 (31.6)
Main clinical discussions topics^a^ n= 501^b^	Monitoring exams Hormone therapy Clarification of clinical history necessity Mental health Gynecological conditions Cancer screening Other phenotypic interventions Cardiovascular risk prevention	325 (64.9) 249 (49.7) 186 (37.1) 145 (28.9) 18 (3.6) 14 (2.8) 12 (2.4) 7 (1.4)
Follow-up of the discussion n= 101^b^	Follow the suggested recommendation Did not follow the suggested recommendation Partially followed the suggested recommendation	76 (71.7) 17 (^16^) 8 (7.5)
Vulnerability conditions^a^ n= 69^b^	Suicide attempt or ideation Self-harm/hetero aggression Domestic/sexual violence Difficult family acceptance Previous psychiatric hospitalization Sex worker (female trans) Homelessness Deprived of liberty Person with a disability Other phenotype interventions without medical supervision	25 (36.2) 18 (26.1) 12 (17.4) 8 (11.6) 6 (8.7) 5 (7.2) 4 (5.8) 4 (5.8) 2 (2.9) 2 (2.9)
Cancer screening^a^ n= 14^b^	Cervix Breast Prostate	10 (71.4) 3 (21.4) 2 (14.3)

a Multiple-response variables.

b Unreported data are not shown in the table. The sample size (n) for each variable is displayed in the first column below each variable, where applicable.

PHC: primary health care; P2P: provider-to-provider.

TC: teleconsultation.

Among patients under the age of 16 (n = 54), 57.4% of consultations documented a support network and the presence of an accompanying adult over 18 years old. Fourteen were accompanied by their mother, ten by both parents, two by their father, one by their grandmother, and four had no familial support network recorded. All individuals under 16 were referred to specialized reference centers for transgender health care, as required by Brazilian legislation.

## DISCUSSION

In our P2P consultation service, calls were evenly distributed between individuals AFAB and AMAB, with a noticeable increase in consultations over time. The most frequently addressed topics were hormone therapy management and laboratory evaluation. Our findings indicated that many transgender individuals were using hormones without medical supervision.

A similar rise in transgender-specific consultations has been reported in other countries, coinciding with the growing number of people identifying as gender diverse since the 1990s, once considered a rare phenomenon (^4^). In September 2022, TelessaúdeRS launched an e-book on transgender health to support PHC professionals (^23^). Developed in response to rising demand and the increasing need for follow-up care within primary care, this resource addresses the insufficient availability of specialized services to meet the needs of this population. The drop in consultations in 2023 may reflect improved access to updated national guidelines and the gradual expansion of transgender-specific health services in Brazil (^7^,^8^).

Most patients were young, consistent with recent findings showing a shift in the profile of transgender individuals seeking care (^4^). There has been a steady rise in the number of AFAB individuals accessing services, now reaching parity with AMAB individuals, mirroring the balanced gender distribution in this study (^4^). The average age for initiating hormone therapy has also decreased over the past decade, now falling below 30 years (^4^,^6^).

A significant proportion of patients were already using hormone therapy at their initial consultation. Although hormone levels and dosages were not available, over 80% of AMAB individuals were using non-guideline-recommended feminizing therapies without medical supervision, whereas a similar proportion of AFAB individuals were using guideline-recommended testosterone formulations.

These patterns may reflect disparities in access to medications across gender identities (^6^,^24^). Contributing factors may include limited availability of free treatments and preferences for non-guideline-recommended hormones due to perceived faster phenotypic results (^11^,^25^,^26^). These findings underscore the importance of assessing history of (particularly nonprescribed) hormone use during P2P consultations. Mental health and addiction were among the most frequently reported concerns. Suicide attempts or ideation were documented in 3.3% of consultations, which is lower than that reported internationally among transgender individuals before transition, but higher than in the general population (^3^,^27^-^29^). This figure may be underestimated, as not all consultations addressed mental health due to their clinical focus. Only 22.1% of cases indicated that the patient was receiving mental health support. These findings highlight a gap in mental health assessment during P2P consultations and among PHC professionals, indicating a need for improved training. Systematically addressing mental health in future consultations could help identify unmet needs and improve care.

Common concerns among PHC professionals included laboratory monitoring, hormone therapy management, clinical history requirements, and mental health issues. With adequate training in gender-affirming care (e.g., mental health assessment and hormone therapy initiation) PHC providers can safely manage more cases without the need for specialist referral. Although international guidelines recommend multidisciplinary specialist follow-up for all transgender patients (^23^,^30^-^32^), P2P consultations can help prioritize referrals, ensuring in-person care is reserved for complex cases, such as individuals pursuing surgery or presenting with severe mental or clinical health conditions. This approach may improve care efficiency and reduce waiting times, which remain prolonged in many settings (^9^,^33^).

Regarding younger patients, the average age of self-identification was 12 years. Notably, over 40% of P2P consultations for individuals under 16 did not document family assistance, which is concerning. This may reflect two non-mutually exclusive possibilities: either insufficient attention to this issue by PHC professionals and consultants, or a genuine absence of family support. Family involvement is known to reduce suicidal ideation, lower depression rates, and improve overall quality of life among transgender individuals (^27^,^34^). Future remote consultation initiatives should systematically address this factor, particularly for younger populations.

### Strengths

To our knowledge, this is the first study to examine how Brazil’s PHC system addresses the needs of transgender individuals using real-world data. The large and diverse sample provides insight into service use across multiple regions, reflecting the broad reach of the TelessaúdeRS platform. In addition, the data are contemporary and mirror recent trends in transgender care delivery. Collectively, these elements represent a novel contribution, offering comprehensive and timely perspective on the specific demands and gaps faced by PHC professionals managing transgender health within the Brazilian public health system.

### Limitations

First, the data were drawn exclusively from PHC professionals who voluntarily used the P2P service, representing a self-selected sample that may not fully reflect the routine practice of all Brazilian PHC providers. Nevertheless, while this limits generalizability at the national level, it provides a high-fidelity, “real-world” perspective on the clinical complexities and support needs of those actively engaged in transgender care. Additionally, the majority of consultations (88.6%) originated from Rio Grande do Sul, the state where the service is based; this requires caution when extrapolating findings nationally but offers a unique “sentinel” perspective on the clinical demands encountered by engaged providers within the public system. Finally, patient data were not stratified by race or skin color due to incomplete reporting, which constrained exploration of these specific social determinants in transgender care.

Furthermore, the extent of information collected varied by consultation, as some data were omitted when not directly relevant to the clinical inquiry. Future research should assess healthcare professionals’ knowledge and preparedness to care for transgender individuals, as well as explore how transgender people perceive the quality, safety, and respectfulness of care received in primary health services. Studies evaluating and guiding improvement of public health policies within the Brazilian Unified Health System are likewise essential to ensure they effectively address the needs of this population. Such evidence is crucial for promoting more inclusive, competent, and equitable care in primary health settings.

In conclusion, this study highlights the increasing volume of clinical questions related to follow-up and hormone therapy for transgender individuals in PHC. It underscores the need to support PHC professionals, particularly regarding hormone therapy initiation and monitoring, mental health care, and support for youth. Ultimately, P2P consultations may enhance PHC professionals’ capacity to deliver appropriate care to transgender patients and facilitate timely access within primary or specialized services. Although our findings reflect the experience of a major telehealth service in southern Brazil and thus have limited national generalizability due to selection bias and regional concentration, they offer a foundational real-world perspective on clinical demands within the Brazilian public health system. Consequently, these results provide a vital evidence base for informing the expansion of similar support strategies and inclusive health policies in other Brazilian regions.

## Data Availability

datasets related to this article will be avail-able upon request to the corresponding author.

## References

[1] GOODMAN M, ADAMS N, CORNEIL T, KREUKELS B, MOTMANS J, COLEMAN E. SIZE AND DISTRIBUTION OF TRANSGENDER AND GENDER
NONCONFORMING (2019). POPULATIONS: A NARRATIVE REVIEW. ENDOCRINOL METAB CLIN NORTH AM.

[2] SPIZZIRRI G, EUFRáSIO R, LIMA MCP, DE CARVALHO NUNES HR, KREUKELS BPC, STEENSMA TD, ET AL. (2021). PROPORTION OF PEOPLE IDENTIFIED AS TRANSGENDER AND NON-BINARY
GENDER IN BRAZIL.. SCI REP.

[3] RENNER J, BLASZCYK W, TäUBER L, DEKKER A, BRIKEN P, NIEDER TO. (2021). BARRIERS TO ACCESSING HEALTH CARE IN RURAL REGIONS BY
TRANSGENDER, NON-BINARY, AND GENDER DIVERSE PEOPLE: A CASE-BASED SCOPING
REVIEW. FRONT ENDOCRINOL.

[4] LEINUNG MC, JOSEPH J. CHANGING DEMOGRAPHICS IN TRANSGENDER INDIVIDUALS SEEKING
HORMONAL (2020). THERAPY: ARE TRANS WOMEN MORE COMMON THAN TRANS
MEN?. TRANSGENDER HEALTH.

[5] FERREIRA MJ, CASTEDO JL, MOTA M, CARVALHO D. (2022). CHARACTERIZATION OF A TRANSGENDER POPULATION IN
PORTUGAL.. ANN ENDOCRINOL.

[6] ITTIPHISIT S, AMPONNAVARAT S, MANABORIBOON N, KORPAISARN S. (2022). THE REAL-WORLD CHARACTERISTICS OF GENDER-AFFIRMING HORMONAL USE
AMONG TRANSGENDER PEOPLE IN THAILAND.. SEX MED.

[7] LUCENA MM, FERREIRA GG, FLOSS M, MELO DAC DE. (2022). SERVIçOS DE ATENDIMENTO INTEGRAL à SAúDE DE
TRANSEXUAIS E TRAVESTIS NO SISTEMA ÚNICO DE SAúDE: UMA
REVISãO INTEGRATIVA. REV BRAS MED FAM E COMUNIDADE.

[8] POPADIUK GS, OLIVEIRA DC, SIGNORELLI MC. (2017). A POLíTICA NACIONAL DE SAúDE INTEGRAL DE
LéSBICAS, GAYS, BISSEXUAIS E TRANSGêNEROS (LGBT) E O ACESSO AO
PROCESSO TRANSEXUALIZADOR NO SISTEMA ÚNICO DE SAúDE (SUS):
AVANçOS E DESAFIOS. CIêNC SAúDE COLETIVA.

[9] BLOSNICH JR, RODRIGUEZ KL, HRUSKA KL, KAVALIERATOS D, GORDON AJ, MATZA A, ET AL. (2019). UTILIZATION OF THE VETERANS AFFAIRS’ TRANSGENDER E-CONSULTATION
PROGRAM BY HEALTH CARE PROVIDERS: MIXED-METHODS STUDY. JMIR MED INFORM.

[10] HOLLAND D, WHITE LCJ, PANTELIC M, LLEWELLYN C. (2024). THE EXPERIENCES OF TRANSGENDER AND NONBINARY ADULTS IN PRIMARY:
CARE: A SYSTEMATIC REVIEW. EUR J GEN PRACT.

[11] SILVA RA DA, SILVA LAV DA, SOARES F, DOURADO I. (2022). USO DE HORMôNIOS NãO PRESCRITOS NA
MODIFICAçãO CORPORAL DE TRAVESTIS E MULHERES TRANSEXUAIS DE
SALVADOR/BAHIA, BRASIL. CIêNC SAúDE COLETIVA.

[12] BELL J, PURKEY E. (2019). TRANS INDIVIDUALS’ EXPERIENCES IN PRIMARY CARE. CAN FAM PHYSICIAN MED FAM CAN.

[13] ROCON PC, WANDEKOKEN KD, BARROS MEB DE, DUARTE MJO, SODRé F. (2019). ACESSO à SAúDE PELA POPULAçãO TRANS
NO BRASIL: NAS ENTRELINHAS DA REVISãO INTEGRATIVA. TRAB EDUC E SAúDE.

[14] TIAN PGJ, HARRIS JR, SEIKALY H, CHAMBERS T, ALVARADO S, EURICH D. (2021). CHARACTERISTICS AND OUTCOMES OF PHYSICIAN-TO-PHYSICIAN TELEPHONE
CONSULTATION: PROGRAMS: ENVIRONMENTAL SCAN. JMIR FORM RES.

[15] LIDDY C, MOROZ I, MIHAN A, NAWAR N, KEELY E. (2019). A SYSTEMATIC REVIEW OF ASYNCHRONOUS, PROVIDER-TO-PROVIDER,
ELECTRONIC CONSULTATION SERVICES TO IMPROVE ACCESS TO SPECIALTY CARE
AVAILABLE WORLDWIDE. TELEMED J E-HEALTH OFF J AM TELEMED ASSOC.

[16] SETTE AL, FRANçOIS P, LESPRIT P, VITRAT V, ROGEAUX O, BREUGNON E, ET AL. (2023). INFECTIOUS DISEASE HOTLINES TO PROVIDE ADVICE TO GENERAL
PRACTITIONERS: A PROSPECTIVE STUDY. BMC HEALTH SERV RES.

[17] CARVALHO RR DE, CARVALHO F, OLIVEIRA EB DE, SILVA RS DA, RADOS DV, MATTIELLO R, ET AL. (2024). DOUBTS ABOUT THE DIAGNOSIS AND TREATMENT OF SYPHILIS IN PREGNANCY
AMONG PRIMARY CARE PROFESSIONALS IN A TELEHEALTH SERVICE.. PLOS ONE.

[18] MEDFORD RJ, GRANGER M, PICKERING M, LEHMANN CU, MAYORGA C, KING H. (2022). IMPLEMENTATION OF OUTPATIENT INFECTIOUS DISEASES E-CONSULTS AT A
SAFETY NET HEALTHCARE SYSTEM. OPEN FORUM INFECT DIS.

[19] ADAMS TCE, LIM CT, HUANG H. (2022). THE PRACTICE OF PSYCHIATRIC E-CONSULTATION: CURRENT STATE AND
FUTURE DIRECTIONS. HARV REV PSYCHIATRY.

[20] YAGNIK KJ, SAAD HA, KING HL, BEDIMO RJ, LEHMANN CU, MEDFORD RJ. (2021). CHARACTERISTICS AND OUTCOMES OF INFECTIOUS DISEASES ELECTRONIC
COVID-19 CONSULTATIONS AT A MULTISITE ACADEMIC HEALTH
SYSTEM.. CUREUS.

[21] D’ANGELO AB, ARGENIO K, WESTMORELAND DA, APPENROTH MN, GROV C. (2021). HEALTH AND ACCESS TO GENDER-AFFIRMING CARE DURING COVID-19:
EXPERIENCES OF TRANSMASCULINE INDIVIDUALS AND MEN ASSIGNED FEMALE SEX AT
BIRTH. AM J MENS HEALTH.

[22] VON ELM E, ALTMAN DG, EGGER M, POCOCK SJ, GøTZSCHE PC, VANDENBROUCKE JP, ET AL. (2007). THE STRENGTHENING THE REPORTING OF OBSERVATIONAL STUDIES IN
EPIDEMIOLOGY (STROBE) STATEMENT: GUIDELINES FOR REPORTING OBSERVATIONAL
STUDIES. LANCET LOND ENGL.

[23] MARTINS ACM, OLIVEIRA EB DE, CARVALHO RR DE, RADOS DRV, RIBEIRO FE DE M, COSTA FBP DA, ET AL. (2022). TELECONDUTAS: ATENDIMENTO àS PESSOAS TRANSEXUAIS E TRAVESTIS NA
ATENçãO PRIMáRIA à SAúDE.

[24] EUSTAQUIO PC, DELA CRUZ JDM, ARAñA Y, ROSOS B, ROSADIñO JDT, PAGTAKHAN RG, ET AL. (2023). PREVALENCE OF AND FACTORS ASSOCIATED WITH THE USE OF
GENDER-AFFIRMING HORMONAL THERAPY OUTSIDE THE REFERENCE REGIMEN AMONG
TRANSGENDER PEOPLE IN A COMMUNITY-LED CLINIC IN METRO MANILA, PHILIPPINES: A
RETROSPECTIVE CROSS-SECTIONAL STUDY. BMJ OPEN.

[25] BASSICHETTO KC, PINHEIRO TF, BARROS C, FONSECA PAM, QUEIROZ RSB DE, SPERANDEI S, ET AL. (2024). BODIES OF DESIRE: USE OF NONPRESCRIBED HORMONES AMONG TRANSGENDER
WOMEN AND TRAVESTIS IN FIVE BRAZILIAN CAPITALS (2019-2021). REV BRAS EPIDEMIOL.

[26] NONPRESCRIBED SEX HORMONE USE AMONG TRANS WOMEN: THE COMPLEX
INTERPLAY OF PUBLIC POLICIES, SOCIAL CONTEXT, AND
DISCRIMINATION TRANSGENDER HEALTH.

[27] AUSTIN A, CRAIG SL, D’SOUZA S, MCINROY LB. (2022). SUICIDALITY AMONG TRANSGENDER YOUTH: ELUCIDATING THE ROLE OF
INTERPERSONAL RISK FACTORS. J INTERPERS VIOLENCE.

[28] BLOSNICH JR, BROWN GR, SHIPHERD PHD JC, KAUTH M, PIEGARI RI, BOSSARTE RM. (2013). PREVALENCE OF GENDER IDENTITY DISORDER AND SUICIDE RISK AMONG
TRANSGENDER VETERANS UTILIZING VETERANS HEALTH ADMINISTRATION
CARE.. AM J PUBLIC HEALTH.

[29] WANG Y, PAN B, LIU Y, WILSON A, OU J, CHEN R. (2020). HEALTH CARE AND MENTAL HEALTH CHALLENGES FOR TRANSGENDER
INDIVIDUALS DURING THE COVID-19 PANDEMIC.. LANCET DIABETES ENDOCRINOL.

[30] KER A, FRASER G, LYONS A, STEPHENSON C, FLEMING T. (2020). PROVIDING GENDER-AFFIRMING HORMONE THERAPY THROUGH PRIMARY CARE:
SERVICE USERS. J PRIM HEALTH CARE.

[31] HEMBREE WC, COHEN-KETTENIS PT, GOOREN L, HANNEMA SE, MEYER WJ, MURAD MH, ET AL. (2017). ENDOCRINE TREATMENT OF GENDER-DYSPHORIC/GENDER-INCONGRUENT
PERSONS: AN ENDOCRINE SOCIETY CLINICAL PRACTICE GUIDELINE. J CLIN ENDOCRINOL METAB.

[32] COLEMAN E, RADIX AE, BOUMAN WP, BROWN GR, DE VRIES ALC, DEUTSCH MB, ET AL. (2022). STANDARDS OF CARE FOR THE HEALTH OF TRANSGENDER AND GENDER
DIVERSE PEOPLE, VERSION 8. INT J TRANSGENDER HEALTH.

[33] POTAPOV A, OLAYIWOLA JN, RADIX AE, MEACHER P, SAJANLAL S, GORDON A. (2021). ELECTRONIC CONSULTATIONS AS AN EDUCATIONAL TOOL TO IMPROVE THE
CARE OF TRANSGENDER PATIENTS IN PRIMARY CARE.. J HEALTH CARE POOR UNDERSERVED.

[34] GANDRA LS, OLIVEIRA LMB, SANTOS IM, DUNNINGHAM WA. (2023). AVALIAçãO DA IMPORTâNCIA DA REDE DE APOIO
PSICOSSOCIAL OFERECIDA PELA FAMíLIA E COMUNIDADE àS PESSOAS
TRANSGêNERAS DURANTE TODO CICLO DE VIDA: UMA REVISãO
SISTEMáTICA. REV BRAS NEUROL E PSIQUIATR.

